# Application of next-generation imaging in biochemically recurrent prostate cancer

**DOI:** 10.1038/s41391-023-00711-0

**Published:** 2023-09-07

**Authors:** Judd W. Moul, Neal D. Shore, Kenneth J. Pienta, Johannes Czernin, Martin T. King, Stephen J. Freedland

**Affiliations:** 1 Duke Cancer Institute and Division of Urology, Duke University, Durham, NC USA; 2 Carolina Urologic Research Center, Myrtle Beach, SC USA; 3 Johns Hopkins School of Medicine, Baltimore, MD USA; 4 David Geffen School of Medicine, University of California, Los Angeles, Los Angeles, CA USA; 5 Brigham and Women’s Hospital and Dana-Farber Cancer Institute, Boston, MA USA; 6 Samuel Oschin Comprehensive Cancer Center, Cedars-Sinai Medical Center, Los Angeles, CA USA; 7 Veterans Affairs Medical Center, Durham, NC USA

**Keywords:** Prostate cancer, Prostate cancer

## Abstract

**Background:**

Biochemical recurrence (BCR) following primary interventional treatment occurs in approximately one-third of patients with prostate cancer (PCa). Next-generation imaging (NGI) can identify local and metastatic recurrence with greater sensitivity than conventional imaging, potentially allowing for more effective interventions. This narrative review examines the current clinical evidence on the utility of NGI for patients with BCR.

**Methods:**

A search of PubMed was conducted to identify relevant publications on NGI applied to BCR. Given other relevant recent reviews on the topic, this review focused on papers published between January 2018 to May 2023.

**Results:**

NGI technologies, including positron emission tomography (PET) radiotracers and multiparametric magnetic resonance imaging, have demonstrated increased sensitivity and selectivity for diagnosing BCR at prostate-specific antigen (PSA) concentrations <2.0 ng/ml. Detection rates range between 46% and 50%, with decreasing PSA levels for choline (1–3 ng/ml), fluciclovine (0.5–1 ng/ml), and prostate-specific membrane antigen (0.2–0.49 ng/ml) PET radiotracers. Expert working groups and European and US medical societies recommend NGI for patients with BCR.

**Conclusions:**

Available data support the improved detection performance and selectivity of NGI modalities versus conventional imaging techniques; however, limited clinical evidence exists demonstrating the application of NGI to treatment decision-making and its impact on patient outcomes. The emergence of NGI and displacement of conventional imaging may require a reexamination of the current definitions of BCR, altering our understanding of early recurrence. Redefining the BCR disease state by formalizing the role of NGI in patient management decisions will facilitate greater alignment across research efforts and better reflect the published literature.

## Introduction

Biochemical recurrence (BCR) occurs in 20–50% of patients with prostate cancer (PCa) within 10 years after primary definitive therapy, i.e., radical prostatectomy (RP) or external beam radiation therapy (EBRT) [1, 2]. In general, BCR is defined as a rise in serum prostate-specific antigen (PSA) levels (Table 1) [3–5]. However, PSA is not necessarily cancer-specific and, after definitive treatments, residual or low-level increases in PSA might be due to benign residual prostate tissue remaining in situ, or due to recurrent benign prostate growth after EBRT or other minimally invasive therapies [6]. Furthermore, there is no consensus on the definition of undetectable PSA and the optimal threshold for initiating therapy post-RP [3, 4]. BCR can be a sign of local recurrence (prostate/seminal vesicles) and/or metastases to lymph node, bone, or viscera [7, 8], particularly in high-risk patients [4]. Detecting recurrent PCa in the early, oligometastatic setting, allows the consideration for metastasis-directed therapy (MDT) [9].

**Table 1:** Current imaging guidelines for BCR [3–5, 13].

	EAU/EANM/ESTRO/ESUR/SIOG		AUA/ASTRO/SUO		NCCN		ASCO	
	Post-RP	Post-EBRT	Post-RP	Post-EBRT	Post-RP	Post-EBRT	Post-RP	Post-EBRT
BCR definition	PSA > 0.4 ng/ml and rising	PSA increase of >2 ng/ml over PSA nadir	PSA increase of 0.2 ng/ml and confirmatory value of ≥0.2 ng/ml	PSA increase of >2 ng/ml over PSA nadir	Detectable^a^ PSA that increases on ≥2 confirmatory tests or increases to PSA levels >0.1 ng/ml	PSA increase of >2 ng/ml over PSA nadir	Detectable^a^ PSA with a subsequent rise	PSA increase of >2 ng/ml over PSA nadir
PET/CT or PET/MRI	PSMA PET/CT if PSA > 0.2 ng/ml Fluciclovine PET/CT or choline PET/CT if PSMA PET/CT unavailable and PSA > 1.0 ng/ml	PSMA PET/CT (if available) or fluciclovine PET/CT or choline PET/CT in patients fit for curative salvage treatment	PET/CT is an alternative to conventional imaging or negative upon conventional imaging		^18^F-DCFPyl PSMA or ^68^Ga-PSMA-11 PET/CT or PET/MRI; ^11^C-choline or ^18^F-fluciclovine PET/CT or PET/MRI		PSMA imaging; ^11^C-choline or ^18^F-fluciclovine PET/CT or PET/MRI; ^18^F-NaF PET/CT in patients with negative conventional imaging and candidates for salvage therapy	
mpMRI	No recommendations		No recommendations		mpMRI preferred over CT for pelvic staging		Whole-body MRI (mpMRI not specified)	

*ASCO* American Society of Clinical Oncology, *ASTRO* American Society for Radiation Oncology, *AUA* American Urologic Association, *BCR* biochemical recurrence, *CT* computed tomography, *DCFPyL* 2-(3-{1-carboxy-5-[6-18F-fluoropyridine-3-carbonyl)-amino]-pentyl}-ureido)-pentanedioic acid, *EANM* European Association of Nuclear Medicine, *EAU* European Association of Urology, *ESTRO* European Society for Radiation Oncology, *ESUR EAU* Section of Urological Research, *EBRT* external beam radiation therapy, *mpMRI* multiparametric MRI, *MRI* magnetic resonance imaging, *NCCN* National Comprehensive Cancer Network, *PET* positron emission tomography, *PSA* prostate-specific antigen, *PSMA* prostate-specific membrane antigen, *RP* radical prostatectomy, *SIOG* International Society of Geriatric Oncology, *SUO* Society of Urologic Oncology.

^a^There is no consensus of what threshold PSA value is defined as undetectable.

Imaging patients with suspected BCR offers key information required by a multidisciplinary team of medical oncologists, radiation oncologists, nuclear medicine physicians, pathologists, and urologists to guide clinical management. For decades, conventional imaging techniques, including computed tomography (CT) and technetium-99m (^99m^Tc) bone scintigraphy, have been used for the assessment of clinical progression in BCR. However, these modalities offer a limited evaluation of recurrent disease at low PSA values (<10 ng ml) [10], with a low probability of positive bone scan (4.5%) and CT (14%) in BCR [11].

Next-generation imaging (NGI) technologies may overcome the sensitivity limitations associated with low PSA results and offer improved diagnostic accuracy for identifying smaller tumor foci compared with conventional imaging [12]. NGI technologies are defined as advanced magnetic resonance imaging (MRI), and positron emission tomography (PET) that combine PCa biology with novel radiotracers to detect recurrent disease currently undetectable with conventional imaging techniques [13]. Previous research on the diagnostic and therapeutic implications of NGI technologies in BCR has been promising [14–18]. A comprehensive systematic review of the literature through 2018 confirmed the high detection rate of various NGI modalities for early recurrence at PSA values < 0.5 ng/ml [19]. Importantly, information provided by NGI has influenced current treatment strategies in up to 70% of patients with BCR [20, 21]. In light of these findings, Radiographic Assessments for Detection of Advanced Recurrence III, European and US medical societies have provided specific recommendations on the use of NGI in BCR [3–5, 13, 22].

This narrative review expands upon the previous research and comprehensively examines the current clinical evidence to elucidate whether NGI may help to identify local recurrence/micrometastatic disease in BCR, thus clarifying the historical BCR definition. We will further discuss the application of NGI modalities to clinical practice in the context of latest recommendations by medical societies. A detailed review of treatment options for patients with BCR will be discussed in a companion narrative review.

## Methods

A comprehensive search of PubMed was conducted to identify relevant publications on the role of NGI in the identification of men with BCR and subsequent treatment, with a particular focus on prospective randomized controlled trials. Searches were limited to English-language publications in peer-reviewed journals from January 2018 to May 2023. Additional articles were identified by examining reference lists in all relevant publications. The literature search included the following keywords: ‘prostate neoplasms’; ‘biochemical recurrence’; ‘imaging’. Database searches yielded 214 articles, of which 88 were included in this review after title/abstract screening and full-text selection. The levels of evidence for the included studies are presented in Table S1.

## Results

### Nuclear imaging

PET radiotracers have been increasingly utilized for diagnostic evaluation and guiding MDT in patients with BCR due to their various tracer affinities for metabolic processes that aid in disease detection and targeted therapy [9, 23]. In BCR, both PSA levels and kinetics influence detection rates for PET tracers [24]. However, when PSA concentrations first begin to rise ( < 0.5 ng/ml), detection depends on the histologically-confirmed tumor size and expression of radiotracer target (e.g., prostate-specific membrane antigen [PSMA]), which can be suboptimal, resulting in limited sensitivity [22, 25–27]. Therefore, it is important to evaluate the performance of radiotracers being considered at PSA concentrations <0.5 ng/ml.

Radiotracers that are approved by the Food and Drug Administration (FDA) for use in patients with PCa include carbon 11 (^11^C)-choline, fluorine 18 (^18^F)-sodium fluoride, ^18^F-fluciclovine (Axumin®, Blue Earth Diagnostics, Inc., Oxford, UK), gallium 68 (^68^Ga)-PSMA-11 (institutional use only in US), and 2-(3-{1-carboxy-5-[(6-^18^F-fluoropyridine-3-carbonyl)-amino]-pentyl}-ureido)-pentanedioic acid (^18^F-DCFPyL; PYLARIFY®, Progenics Pharmaceuticals, Inc. North Billerica, MA; US only).

#### Choline

Choline is essential for phospholipid biosynthesis in all cell membranes. In PCa, increased choline uptake by malignant cells with increased cell proliferation can be assessed by ^11^C-choline PET. A PSA value of 1–2 ng/ml is estimated to be the optimal threshold for the diagnostic efficiency of choline PET in BCR [28]. In 358 patients with BCR evaluated with ^11^C-choline PET/CT, the percentage of patients with positive scans increased with increasing PSA levels: 19% of patients with PSA levels of 0.2–1.0 ng/ml, 46% of patients with PSA levels 1–3 ng/ml, and 82% of patients with PSA levels >3 ng/ml [28]. According to a meta-analysis, ^11^C-choline PET/CT has displayed good accuracy in detection of lymph node metastasis and/or distant lesions, but the findings on local recurrence were inconclusive due to high between-study heterogeneity [14]. An additional disadvantage of ^11^C-choline is its short half-life (20 min), limiting availability to centers with a cyclotron/radiochemistry facility onsite [17]. Alternatively, ^18^F-choline has a longer half-life and similar performance as ^11^C-choline in BCR [29]. Increased lesion detection rates have been observed for PSADT of ≤6 months (65%) and average PSA levels >1 ng/ml (67%) [30].

#### Fluciclovine

In PCa, amino acid metabolism is upregulated, explaining the effectiveness of ^18^F-fluciclovine, a synthetic leucine analog radiotracer for detecting BCR. In LOCATE, an open-label, prospective phase 4, multicenter study of 221 patients with PCa, ^18^F-fluciclovine PET/CT positivity rates were proportional to PSA concentrations: detection rates were 31% in patients with PSA levels 0–0.5 ng/ml, 50% in patients with PSA levels >0.5–1.0 ng/ml, and 66% in patients with PSA levels >1.0–2.0 ng/ml [31]. In another prospective study of 89 patients with BCR, ^18^F-fluciclovine PET/CT demonstrated improved detection performance for local, lymph nodal, and bone relapse, in addition to higher sensitivity (37% vs. 32%) and specificity (67% vs. 40%) compared with ^11^C-choline [32]. Additionally, ^18^F-fluciclovine PET/CT demonstrated significantly better sensitivity than ^11^C-choline at PSA concentrations <1 ng/ml (*p* < 0.001). Similar results were reported when ^18^F-fluciclovine was compared with ^18^F-fluorocholine [33].

The open-label FALCON trial of 104 patients who developed a first episode of BCR reported that ^18^F-fluciclovine PET/CT imaging resulted in a change in management for 64% (*n* = 66) of those scanned, 24% of whom transitioned from salvage to systemic therapy [34]. The prospective EMPIRE-1 study used ^18^F-fluciclovine to guide salvage EBRT post-RP in 165 patients with BCR and no evidence of metastases upon conventional imaging; 3-year event-free survival was significantly improved in the ^18^F-fluciclovine PET/CT group (Δ 12.5%; 95% CI 4.3–20.8; *p* = 0.003) compared with the conventional imaging group [35]. Given the approval of fluciclovine by regulatory authorities, this radiotracer is widely available in the US and Europe, but has limited use in the rest of the world due to widespread availability of PSMA-targeted PET imaging radiotracers.

#### Sodium fluoride

^18^F-sodium fluoride (^18^F-NaF) is a bone-specific radiotracer that can identify areas of abnormal osteogenic activity and is used to detect skeletal metastases [36]. According to a per-patient (*N* = 148) and per-lesion (*N* = 744) analysis in patients with PCa, ^18^F-NaF demonstrated superior imaging sensitivity and specificity in detection of bone metastases compared with conventional scintigraphy (*p* < 0.001, for both) [37]. In a prospective study of 37 patients with BCR, the positive detection rate of bone metastases missed by conventional CT and bone scan was 16% [38]. A retrospective analysis observed that mean PSA levels were two-fold higher (4.11 vs. 2.02 ng/ml) in patients positive for bone metastases (22% [8/36]) compared with patients with negative ^18^F-NaF PET/CT scans [39]. Additionally, PSA velocity significantly predicted positive scan outcomes. Initial results from the National Oncologic PET Registry revealed ^18^F-NaF PET imaging (*N* = 1997) revised the treatment plan for 52% of cases where first osseous metastasis were detected [40]. Subsequent analysis from this registry demonstrated that detection of osseous metastases with ^18^F-NaF PET imaging was important for effective patient management and, ultimately, patient survival [41]. However, the reduced specificity and narrow applicability of ^18^F-NaF to bone compared with novel PET tracers, have limited its use [36].

#### PSMA

PSMA is a transmembrane glycoprotein overexpressed in PCa compared with normal prostate tissues and other tissues [42]. PSMA PET has produced encouraging diagnostic results and is an attractive target due to its rapid internalization and blood clearance.

#### ^68^Ga-PSMA

^68^Ga-PSMA PET is a promising diagnostic technique given its ability to detect recurrent PCa. A significant increase (*p* < 0.001) in detection rates across predefined PSA ranges was reported in a single-arm prospective study of 635 patients with BCR imaged with ^68^Ga-PSMA-11 PET/CT or PET/MRI [43]. In this study, the overall detection rate was 75%, with a positive predictive value (PPV) of 0.84-0.92. Based partly on the results of this study, ^68^Ga-PSMA-11 PET was approved by the FDA for institutional use in 2020 for patients with suspected metastasis curable via surgery or radiation therapy, as well as for those with suspected BCR based on elevated PSA values [44]. In a subsequent prospective multicenter study examining ^68^Ga-PSMA PET in 2005 patients with recurrent PCa, the overall per-patient scan positivity rate was 78%, with increasing positivity rates at higher PSA concentration subgroups: <0.25 ng/ml, 44.8%; 0.25-0.49 ng/ml, 50.5%; 0.5–0.99 ng/ml, 69.2%; 1.00-1.99 ng/ml, 78.1%; and >2.00 ng/ml, 95% (95% CI 92–97) [45]. Factors that significantly correlated with the detection rate included Gleason grade group from RP biopsies (*p* < 0.001) and clinical T-stage (*p* < 0.01), but not Gleason grade group at initial biopsy (*p* = 0.86). Confirmed by histopathology, ^68^Ga-PSMA-11 PET imaging reported PPV of 83% in bone, 83% in prostate and prostate bed, 72% in pelvic lymph nodes, and 88% in extrapelvic soft tissues. Furthermore, in a prospective study, ^68^Ga-PSMA-11 PET impacted staging and management of 197 patients with BCR [46]. A prospective phase 3 study of 82 patients demonstrated per-patient positivity that was noninferior when PSMA-11 was labeled with ^18^F or ^68^Ga [47].

In a prospective, direct comparison trial of ^18^F-fluciclovine PET/CT and ^68^Ga-PSMA PET/CT for patients with post-operative PSA levels 0.2–2.0 ng/ml, PSMA PET/CT detected recurrence sites at lower PSA concentrations more frequently and with high inter-reader agreement compared with ^18^F-fluciclovine PET/CT [48]. Overall, the detection rate was 26% for ^18^F-fluciclovine PET/CT and 56% for PSMA PET/CT. However, in a study of patients with BCR (mean PSA, 14.9 ng/ml), the detection rate for ^68^Ga-PSMA-11 PET was significantly reduced compared with ^18^F-fluciclovine for local recurrence near the urinary bladder (28% vs. 38%; *p* = 0.03) [49].

#### DCFPyL

The performance of ^18^F-DCFPyL, a second-generation PSMA radiotracer, was similar to ^68^Ga-PSMA-11 in a direct comparison [50]. The phase 2/3 OSPREY trial of 93 patients with BCR by conventional imaging demonstrated high sensitivity (median, 96%; 95% CI 88–99) and PPV (median, 82%; 95% CI 74–90) for ^18^F-DCFPyL PET/CT [51]. Sensitivity and PPV for ^18^F-DCFPyL PET/CT ranged from 89–100% and 62–89%, respectively, in patients with low PSA values (<2 ng/ml). In another phase 2 study (*N* = 92), similar PPV values (89%; 95% CI 75–97) were reported [52]. In the CONDOR phase 3 study of 208 patients with suspected BCR (median PSA, 0.8 ng/ml) and negative or equivocal upon conventional imaging, ^18^F-fluciclovine or ^11^C-choline PET, imaging with ^18^F-DCFPyL had a disease detection rate and correct localization rate (CLR) of 59–66% and 85–87%, respectively, by independent blinded review. ^18^F-DCFPyL PET results also changed the clinical management in 64% of patients, including 21% of patients who had negative findings with conventional imaging [53]. Of note, the median CLR was 73% for patients with a baseline PSA level of <0.5 ng/ml. Multivariable analysis from two studies of 245 patients with BCR demonstrated that PSA levels and PSA doubling time were independent predictors of scan positivity and disease location [54]. In 2021, ^18^F-DCFPyL received FDA approval as a diagnostic PET radiotracer for PSMA-positive lesions in patients with PCa and suspected metastases who are candidates for definitive therapy or with BCR.

#### Investigational radiotracers

The radiohybrid (rh)PSMA tracers were designed to address limitations observed with ^68^Ga-PSMA PET radiotracers, including bladder and urethra accumulation, which potentially interfere with the diagnosis of localized BCR [55, 56]. The lead compound in a new class of PSMA radiotracers, ^18^F-rhPSMA-7, has demonstrated rapid blood clearance and low bladder retention in preclinical studies [57]. A key advantage is the long half-life of fluorine radiotracers (110 min) [58]. ^18^F-rhPSMA-7.3 is under consideration for FDA approval based partly on the results of the phase 3, prospective, multicenter SPOTLIGHT trial (NCT04186845). For men (*N* = 366) with BCR and median (range) PSA, 1.27 (0.03–134.6) ng/ml, the patient-level correct detection rate (CDR; both conventional imaging and histopathology) was 56.8% (95% CI 51.6–62.0) [59]. In a subgroup of patients whose disease was confirmed by histopathology only, the patient-level CDR was high (81.2%, 95% CI 69.9–89.6). In addition, detection rates improved with increasing PSA levels: <0.5 ng/ml, 64%; ≥0.5–0.99 ng/ml, 76%; ≥1.0–1.99 ng/ml, 93%; ≥2.0–4.99 ng/ml, 96%; ≥5.0–9.99 ng/ml, 88%; ≥10 ng/ml, 100%.

Another radiotracer under investigation, ^18^F-PSMA-1007, has the advantage of being cleared via hepatobiliary excretion [60]. A prospective, phase 3 multicenter study (*N* = 190; NCT04102553) demonstrated that ^18^F-PSMA-1007 PET/CT was superior to ^18^F-fluorocholine PET/CT [61]. A positive CDR of 94% (*n* = 179/190) was determined by three independent readers and confirmed by an independent expert panel. For ^18^F-PSMA-1007, CDR were 0.82 (95% CI 0.78-0.86) and 0.77 (95% CI 0.72-0.82) for positive or negative malignancy, respectively, and were statistically superior to 0.65 (95% CI 0.60-0.71) and 0.57 (95% 0.51-0.62) for ^18^F-fluorocholine, respectively (*p* < 0.001). Similar to other PSMA radiotracers, the CDR for ^18^F-PSMA-1007 increased with increasing PSA levels. Subsequent to imaging, diagnostic thinking was changed in 62% (*n* = 93/149) of patients.

### PSMA PET and BCR risk stratification

Accumulating evidence suggests that the detection performance of PSMA-targeted PET imaging varies across BCR risk categories as defined by the EAU risk-scoring system [62–64]. Post-RP, high-risk is defined as PSADT ≤ 1 year or Gleason score 8–10; post-EBRT, high-risk is defined as interval from primary therapy to biochemical failure ≤18 months or Gleason score 8–10 [64]. In a multivariate analysis of patients with BCR and no known metastasis (*N* = 1960), the BCR high-risk group had a higher likelihood of metastatic disease by PSMA PET compared with the low-risk group (odds ratio, 2.91; 95% CI 2.18–3.93) [62]. Among patients with high-risk BCR, PSMA PET positivity rate for distant metastases was higher post-EBRT (70% [110/158]) compared with post-RP (37% [342/931]). Thus, EAU BCR risk groups do not completely characterize the extent of disease. However, PSMA PET can provide key information to refine disease extent, and potentially inform treatment decisions. In this context, a retrospective single-center study of 276 men with detectable PSA levels following EBRT or brachytherapy who underwent ^68^Ga-PSMA PET/CT found positive scans in 55 of 73 patients (75.3%) with pre-scan PSA values below the Phoenix definition of BCR, <0.5 ng/ml (66.7% [8/12]); 0.5‒ < 1.0 ng/ml (77.8% [14/18]); 1‒ < 2.0 ng/ml (76.7% [33/43]). In this subgroup, 38/73 (52.1%) patients were identified as the suitable candidates for salvage therapy based on the PSMA-detected local recurrence and/or nodal disease within the extended pelvic lymph node dissection field [65]. Notably, a panel of cancer specialists at the Advanced Prostate Cancer Consensus Conference 2019 rated PSMA PET as the preferred imaging modality for detecting clinical progression (80–87% consensus), and for confirming a CT/scintigraphy-based diagnosis of oligorecurrent oligometastatic PCa (75% consensus) in patients with BCR [66].

### NGI-guided early intervention after primary definitive therapy

PET radiotracers have shown promise in identifying patients with BCR or oligometastatic PCa who would benefit from early intervention post-RP or post-EBRT, including MDT. In the phase 2 STOMP study, patients (*N* = 62) with BCR and ≤3 extracranial metastatic lesions (by choline PET/CT) were randomized to surveillance or MDT (surgery or stereotactic body radiotherapy [SBRT]) [67]. Median androgen deprivation therapy (ADT)-free survival for MDT compared with surveillance at 5 years (34% vs. 8%; hazard ratio [HR] 0.57; 80% CI 0.38-0.84; *p* = 0.06) confirmed the results at 3 years (HR 0.60; 80% CI 0.40-0.90; *p* = 0.11), but neither time point achieved statistical significance [67, 68]. In another study, patients (*N* = 33) with BCR and ≤3 extracranial metastatic lesions (by ^18^F-NaF PET/CT, conventional CT, and bone scan) were treated with MDT (SBRT) [69]. In this study, the 1- and 2-year local progression-free survival (PFS) rates were 97% (95% CI 91–100) and 93% (95% CI 84–100), while distant PFS were 58% (95% CI 43–77) and 39% (95% CI 25–60), respectively. Furthermore, quality of life was maintained [69].

In the ORIOLE phase 2 study, 54 patients with oligometastatic disease on conventional imaging were randomized to SBRT or observation [70]. All patients treated with SBRT had a baseline and post-treatment PSMA scan. The investigative team and patients were blinded to the PSMA PET data; therefore, for some patients, baseline PET lesions were not included in the treatment fields. The results demonstrated that SBRT was associated with improved outcomes at 6 months. Treatment of all lesions identified using ^18^F-DCFPyL-PSMA PET/CT with MDT (all lesions treated, *N* = 19; lesions untreated, *N* = 16), was associated with improved median PFS at 6 months (treated, not evaluable; untreated lesions, 11.8 months; *p* = 0.006) and median distant metastases-free survival (treated, 29 months; untreated lesions, 6.0 months; *p* < 0.001) [70].

Similarly, in a prospective phase 2 study including 72 patients post-EBRT or RP with rising PSA (0.4–3.0 ng/ml) and negative upon conventional imaging, 53% (*n* = 38) had oligorecurrent disease following ^18^F-DCFPyL PET whole-body MRI/CT and, due to NGI, were treated with MDT without ADT [71]. With a median follow-up of 15.9 months posttreatment, the overall response rate was 60% (22/37), including 22% (8/37) with no evidence of disease for a median duration of 7.7 months. However, the long-term prognosis of these patients remains unclear. In comparison, EMPIRE-1 trial demonstrated that treatment decision and EBRT planning guided by ^18^F-fluciclovine PET/CT plus conventional imaging (*n* = 79) versus conventional imaging alone (*n* = 81) significantly improved 4-year failure-free survival rate in patients with detectable post-RP PSA and negative conventional imaging (75.5% vs 51.2%; *p* < 0.0001) [35]. A retrospective analysis of 305 patients with BCR detected with ^68^Ga-PSMA PET treated with MDT (median nodal 1 [range 0–19]; median extranodal 1 [range 0–5]) plus ADT demonstrated that the MDT + ADT combination significantly improved biochemical PFS (*p* < 0.001) [72]. The significant increase was only reported in men receiving MDT + ADT for >6 months. However, even though ADT + MDT in combination improved biochemical PFS significantly, the investigators noted that disease progression occurred significantly more often with MDT monotherapy patients (85% vs. 29%; *p* < 0.001) requiring additional salvage therapies compared with ADT + MDT combination [72]. Of note, following MDT, 95% of patients experienced a PSA reduction with or without concurrent ADT.

Ongoing clinical trials will help elucidate the long-term survival benefits of NGI-directed therapeutic interventions in patients with BCR or oligometastatic disease (Table 3).

### Whole-body mpMRI

Whole-body multiparametric MRI (mpMRI) is characterized by superior resolution of anatomy and soft tissue, making it highly sensitive for local recurrences. mpMRI involves advanced sequences, including assessment of Brownian motion of water molecules within tissue termed diffusion-weighted imaging (DWI) and dynamic contrast enhancement (DCE) imaging, which assesses vascular perfusion of tissue [21]. Studies suggest that DCE may be more effective at detecting BCR. In a study of 60 patients with BCR evaluated by DCE, DWI, and three-dimensional magnetic resonance spectroscopy (MRS), sensitivities were 100%, 71%, and 54% for DCE, DWI, and MRS post-RP (*N* = 28; median PSA, 5.8 ± 2.2 ng/ml), and 97%, 97%, and 78% post-EBRT (*N* = 32; median PSA 13.5 ± 3.2 ng/ml), respectively [73].

Combinations of various mpMRI techniques have also been investigated. In a study of 43 patients with post-RP BCR (mean PSA level, 0.71 ng/ml), combination mpMRI, i.e., T2-weighted imaging combined with DWI (*p* = 0.04), DCE-MRI (*p* = 0.02), or both (*p* < 0.001), was more predictive of local recurrence compared with T2-weighted MRI alone [74]. A meta-analysis of mpMRI studies post-RP showed that the highest pooled mean sensitivities were demonstrated by DCE + MRS (89%), followed by DWI + T2 imaging (82%), and DCE + T2 imaging (82%) [75]. The DCE + MRS and DCE + T2 imaging combinations also reported the highest pooled mean specificities (92%). Most studies included in this analysis evaluated local recurrence with PPV that varied depending on the sequence. Data indicate that combination mpMRI may be the most effective in detecting BCR among mpMRI modalities, and further investigation is warranted. Prospective data also suggest a potential role for mpMRI to guide salvage high-intensity focused ultrasound (sHIFU) in patients with BCR post-EBRT, evaluate local recurrence prior to and following the sHIFU, and inform subsequent treatment decisions [76].

### NGI in clinical practice and PCa guidelines

Advanced imaging modalities could contribute to guiding subsequent treatment decision-making in patients with BCR. However, several obstacles could prevent the routine adoption of NGI in the US clinical practice, including racial, geographical, and insurance coverage disparities in access [77, 78]. In contrast, due to the actions taken by regulatory authorities in other countries such as Australia, accessible and insurance-covered PSMA PET/CT is replacing conventional imaging as the preferred imaging modality for patients with BCR, with potentially beneficial impact on patient care optimization [79]. Lack of specificity (e.g., uptake in other benign or malignant lesions) for PCa associated with fluciclovine and PSMA PET may result in false-positive lesions, highlighting the importance of a concurrent diagnostic CT scan [26, 80]. Additional reasons for false-positive findings include low standardized uptake values, post-EBRT activity and inflammation and challenges with analysis around the bladder neck [81, 82]. Other limitations with PSMA PET include low resolution for lesions <4 mm and low target expression (Table 2) [81]. Limited global availability is another limitation of PSMA PET. “Flares” in PSMA tracer uptake, e.g., after commencing ADT, may result in increased sensitivity of existing disease, if confirmed, and complicate treatment choice [83, 84]. An expert panel published guidelines to standardize interpretation of PSMA PET that should improve accuracy, precision, and repeatability [85].

**Table 2:** Potential reasons for false-positive and false-negative findings with PSMA PET [52, 81, 82].

False-positive findings	False-negative findings
Low SUV_max_^a^	Adjacent bladder activity
Non-specific ligand uptake	Lack of or low PSMA expression
Post EBRT residual activity	Small metastases
Inflammation	

*EBRT* external beam radiation therapy, max maximum, *PET* positron emission tomography, *PSMA* prostate-specific membrane antigen, *SUV* standardized uptake values.

^a^Threshold not clearly defined*.*

Optimal results with mpMRI are largely dependent on the equipment, acquisition of high-quality images, and the experience of the radiologist interpreting the images [86]. In addition, treatment with ADT has been reported to negatively impact the sensitivity and accuracy detection by mpMRI [87]. To address these issues, the Prostate Imaging for Recurrence Reporting was published to globally standardize parameters for image acquisition, image interpretation, and reporting of mpMRI in local pelvic PCa recurrence after primary treatment [88].

Current guidelines from medical societies afford clinicians the opportunity to consider imaging modalities under certain circumstances (Table 1) [3–5, 13]. However, clinical evidence is needed to recommend the most appropriate imaging technique available to address the clinical issue in question with the highest level of accuracy and confidence [4]. The AUA/ASTRO/SUO guidelines advise that clinicians utilize PET/CT as an alternative to conventional imaging, or when detection of foci suspicious for malignancy is interpreted as negative or equivocal on conventional imaging in patients at high risk for metastases [3]. The ASCO guideline recommends PSMA imaging; ^11^C-choline or ^18^F-fluciclovine PET/CT or PET/MRI; ^18^F-NaF PET/CT and/or whole-body MRI in patients with BCR and negative conventional imaging who are candidates for salvage therapy [13]. In contrast, the 2023 National Comprehensive Cancer Network Guidelines® recommend that PSMA PET tracers serve as front-line imaging tools for BCR due to the increased sensitivity and specificity compared with conventional imaging [4]. The National Comprehensive Cancer Network also suggests that mpMRI is preferred over CT for pelvic staging of BCR [4]. Additionally, the Radiographic Assessments for Detection of Advanced Recurrence VII working group recommends considering molecular-targeted imaging, a new term suggested for NGI, to detect metastatic foci and inform subsequent treatments in patients with rising PSA ≥ 0.2 ng/dl after primary treatment, including patients with PSA levels below the Phoenix definition [89].

## Discussion

NGI for BCR is changing the evaluation and management of recurrent PCa. In the near future, BCR identified by conventional imaging will be replaced in clinics with access to NGI. Multiple novel radiotracers are being evaluated clinically (Table 3), thus, as NGI becomes more sensitive for detecting recurrent disease, and is accessible for more patients, the current definition of the disease state for BCR will need to evolve to address the influence of NGI on BCR diagnosis. In other words, NGI can identify recurrence at lower PSA levels compared with conventional imaging, thus affecting treatment selection, and allowing novel interventional strategies that may enhance patient outcomes (Table 4). However, it is necessary for the medical community to align on an updated definition for BCR that incorporates the role of NGI. Therefore, we propose that it is time for the disease state of BCR to be updated and redefined to account for the impact of NGI.

**Table 3:** Active clinical trials evaluating NGI in patients with BCR.

Study Phase	Anticipated Enrollment	Primary Objective	Primary Outcome Measure(s)	Estimated Completion Date	Study Identifier
1/2	60	Assessment of performance of ^68^Ga-P16-093 in intermediate to high-risk patients with primary PCa or BCR	Sensitivity Proportion of patients with treatment change due to lesion detection	July 2022	NCT03444844
3	217	Diagnostic performance and safety of ^18^F-DCFPyL vs ^18^F-fluorocholine in patients with BCR post-RP	Per-patient detection rate of ^18^F-DCFPyL PET/CT vs. ^18^F-fluorocholine PET/CT across 10 weeks	February 2022	NCT04734184
3	136	Diagnostic performance and safety of ^18^F-PSMA-1007 PET/CT in patients with BCR	Region-level PPV; Patient-level CDR	August 2023	NCT04742361
2	100	Assessment of ^18^F-DCFPyL PET/CT to identify early oligometastatic PCa in patients with BCR	Detection rate and performance metrics of ^18^F-DCFPyL PET/CT	September 2023	NCT03160794
3	190	Assessment of the diagnostic performance of ^18^F-CTT1057 for detection and localization of PSMA-positive lesions in patients with PCa diagnosed with BCR post-RP or EBRT using CTS as reference 1:1 Random sequence: PET/CT imaging with ^18^F-CTT1057 followed by ^68^Ga-PSMA-11 or vice versa	CLR, defined as the proportion of regions containing ≥1 TP lesion PPV, defined as proportion of patients who have one true positive lesion	December 2023	NCT04838613
Pilot [93]	100	Comparison of ^68^Ga-PSMA-11 and ^18^F-PSMA-1007	Patient-level detection rate	December 2023	NCT05079828
3 [94]	193	Evaluation of the success rate for EBRT for BCR post-RP with ^68^Ga-PSMA-11 vs. standard of care	Success rate of salvage EBRT defined as BPFS after initiation of EBRT	July 2024	NCT03582774
2	196	Evaluation of MDT + WPRT + ADT vs. MDT + ADT in patients with BCR and PSMA PET-detected nodal recurrence	MFS	April 2025	NCT03569241
2/3	464	Evaluation of systemic therapy + PET-directed local therapy vs. systemic therapy only in patients with BCR post RP or EBRT and 1–5 suspicious lesions	CRPC-free survival	December 2025	NCT04787744
2	140	Comparison of ^68^Ga-PSMA-11 and ^18^F-fluciclovine to inform radiotherapy decision-making in patients with detectable PSA post RP	DFS	December 2025	NCT03762759
2/3	130	Evaluation of the success rate for EBRT with ^18^F-DCFPyL 11 vs. standard of care in patients with BCR, localized high-risk PCa or OMPC on conventional imaging	FFS	April 2026	NCT03525288
3	873	Evaluation of MDT + 6-mo ADT + enzalutamide vs MDT + 1-mo ADT vs MDT alone in patients with BCR and positive PSMA PET/CT or PET/MRI for oligorecurrent disease	PMFS	April 2032	NCT05352178
3	804	Application of ^18^F-fluciclovine PET/CT in delivery of treatment to patients with BCR	PFS assessed up to 10 years	December 2032	NCT04423211

*ADT* androgen deprivation therapy*, BCR* biochemical recurrence, *BPFS* biochemical progression-free survival, *CDR* correct detection rate; *CLR* correct localization rate, *CRPC* castration-resistant prostate cancer, *CT* computed tomography, *CTS* composite truth standard, *DCFPyL* 2-(3-{1-carboxy-5-[6-18F-fluoropyridine-3-carbonyl)-amino]-pentyl}-ureido)-pentanedioic acid, *DFS* disease-free survival, *EBRT* external beam radiation therapy, *FFS* failure-free survival, *MDT* metastasis-directed therapy, *MFS* metastasis-free survival, *MRI* magnetic resonance imaging, *NGI* next-generation imaging, *OMPC* oligometastatic prostate cancer, *PCa* prostate cancer, *PET* positron emission tomography, *PFS* progression-free survival, *PMFS* polymetastases-free survival, *PPV* patient-level positive predictive value, *PSA* prostate-specific antigen*, PSMA* prostate-specific membrane antigen, *RP* radical prostatectomy, *TP* true positive; *WPRT* whole pelvis radiotherapy.

**Table 4:** FDA-approved PET radiotracers for BCR in PCa [28, 31, 32, 34, 35, 37, 40, 45, 46, 51, 53, 67, 69–72].

Characteristics	^11^C-Choline	^18^F-Fluciclovine	^18^F-NaF (bone-specific)	^68^Ga-PSMA	^18^F-DCFPyl-PSMA
Half-life, min	20	110	110	68	110
Detection rate, % (PSA level, ng/ml)	19 (0.2–1); 46 (1–3); 82 ( > 3)^a^	31 (0–0.5); 50 ( > 0.5–1); 66 ( > 1–2)	NA	45 ( < 0.25); 51 (0.25–0.49) 69 (0.5–0.99) 78 (1.0–1.99); 90 (2.0–4.99); 93 (5.0–9.99) 96 ( ≥ 10)	36 ( < 0.5); 51 (0.5–0.99); 67 (1.0–1.99); 85 (2.0–4.99); 97 ( ≥ 5)^b^
Specificity, % (95% CI)	89 (73–93)^c^	67	90 (86–93)^d^	NA	NA
Sensitivity, % (95% CI)	89 (83–93)^c^	37	98 (95–99)^d^	NA	96 (88–99)
PPV, %^e^	NA	97	NA	82 ^f^	82 (74–90)
Tissue-specific performance			NA		
Detection rate, % (95% CI)					
Bone	25 (16–34)	100^e^		83	63 (43–82)^b,e^
Local	27 (16–38)	100^e^		83	80 (67–92)^b,e^
Lymph node, pelvic	36 (22–50)^g^	91^e^		72	80 (59–83)^b,e^
Visceral				88	29 (7.6–65)^b,e^
Changed clinical management, %	NA	64	52	57	64
Impact on patient outcomes in prospective clinical trials	Improved 3- and 5-year ADT-free survival posttreatment with MDT compared with surveillance	Improved 3-year event-free survival	Improvement in local and distant PFS post-MDT; Maintained QoL	Significant improvements in biochemical PFS in patients who received MDT + ADT for >6 months	Improved median PFS at 6 months post-MDT; 60% response rate at 16 months post-MDT

*BCR* biochemical recurrence, ^*11*^*C* carbon 11, ^*18*^*F-DCFPyL* 2-(3-{1-carboxy-5-[6-18F-fluoropyridine-3-carbonyl)-amino]-pentyl}c-ureido)-pentanedioic acid, ^*18*^*F* fluorine 18, ^*68*^*Ga* gallium 68, *ADT* androgen deprivation therapy, *BCR* biochemical recurrence, *CI* confidence interval, *EBRT* external beam radiation therapy, *FDA* Food and Drug Administration, *MDT* metastasis-directed therapy, *NA* not available, *NaF* sodium fluoride, *PCa* prostate cancer, *PET* positron emission tomography, *PFS* progress-free survival, *PSMA* prostate-specific membrane antigen, *PPV* positive predictive value, *PSA* prostate-specific antigen, *QoL* quality of life, *s* salvage.

^a^Values are percent positivity [95].

^b^Median values from three independent, blinded, board-certified nuclear medicine physicians.

^c^Meta-analysis using a bivariate model of data from pooled studies [96].

^d^Meta-analysis from 12 pooled studies.

^e^PPV = Number of true positives/(number of true positives + number of false-positives).

^f^PPV confirmed by histopathology [45].

^g^Detection rate for lymph node and distant metastases are combined.

**Fig. 1:**
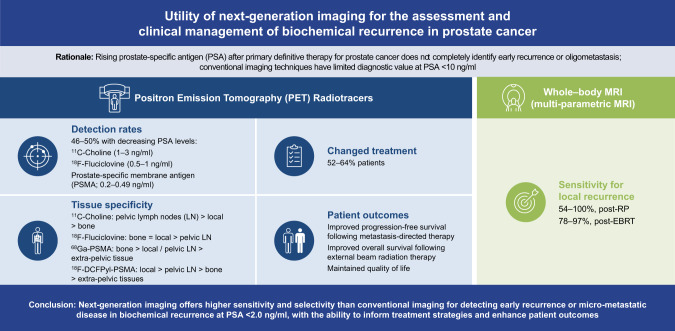
Utility of next-generation imaging for the assessment and clinical management of biochemical recurrence in prostate cancer [14, 28, 31, 32, 34, 35, 43, 46, 53, 67, 70, 72, 73, 97].

Advances in MRI and PET have demonstrated the potential to detect BCR not otherwise captured by increases in PSA concentrations and conventional imaging (Fig. 1). At the time of this review, mpMRI and PSMA PET have demonstrated the highest sensitivity and specificity of NGI applications. Of note, the performance of PSMA PET radiotracers has been evaluated in multiple sites of recurrence, in contrast to mpMRI, which focused on local recurrences. Importantly, application of NGI is beneficial only if it informs clinical management decisions that lead to a more favorable clinical outcome. A novel area of research is the application of mpMRI to the “radiomics” of PCa, i.e., extraction and quantitative assessment of advanced imaging features of prostate tumors, including volume/shape, volume intensity histograms, texture, and transform analysis to identify subregions with distinct phenotypic characteristics [90]. This radiomic approach could facilitate earlier detection and more personalized patient management.

Clinical nomograms are being developed to identify patients who should be considered for NGI [54, 91]. Unfortunately, limited data exist on the application of NGI to BCR, specifically how NGI influences the timing of intervention and subsequent patient outcomes. However, clinical studies indicate that utilizing NGI to identify patients with BCR who would benefit from treatment, can have an impact on patient outcomes [35, 70]. However, limitations remain associated with image resolution and their routine adoption in clinical practice [83]. Despite the evidence of cost-effectiveness in the BCR setting [92], the wider use of NGI in the US practice is further impeded by inconsistent access and insurance coverage issues [78]. Clearly, there is a need for more evidence-based prospective clinical trials [35, 70] and correlative guidelines to support the routine application of NGI in patients with BCR. Nevertheless, NGI is here to stay and use will only increase with time.

The present narrative review did not follow a systematic search strategy based on the Preferred Reporting Items for Systematic Reviews and Meta-Analyses framework to allow for a broad coverage of the evidence on the rapidly evolving role of NGI technologies in BCR management. This limitation should be considered when interpreting the synthesized evidence in this review.

## Conclusion

Current evidence confirms the increased sensitivity and selectivity of NGI technologies for detecting BCR, with the potential to inform treatment strategies. Global clinical practice guidelines recommend NGI in the diagnostic workup of patients with BCR upon negative/equivocal findings or as an alternative to conventional imaging. Considering the advancements observed with NGI, we propose that the disease state of BCR needs to be updated and redefined.

### Supplementary information

Supplementary Appendix
