# Correction: Application of next-generation imaging in biochemically recurrent prostate cancer

**DOI:** 10.1038/s41391-024-00791-6

**Published:** 2024-01-17

**Authors:** Judd W. Moul, Neal D. Shore, Kenneth J. Pienta, Johannes Czernin, Martin T. King, Stephen J. Freedland

**Affiliations:** 1 Duke Cancer Institute and Division of Urology, Duke University, Durham, NC USA; 2 Carolina Urologic Research Center, Myrtle Beach, SC USA; 3 Johns Hopkins School of Medicine, Baltimore, MD USA; 4 David Geffen School of Medicine, University of California, Los Angeles, Los Angeles, CA USA; 5 Brigham and Women’s Hospital and Dana-Farber Cancer Institute, Boston, MA USA; 6 Samuel Oschin Comprehensive Cancer Center, Cedars-Sinai Medical Center, Los Angeles, CA USA; 7 Veterans Affairs Medical Center, Durham, NC USA

**Keywords:** Prostate cancer, Prostate cancer

Correction to: *Prostate Cancer and Prostatic Diseases* 10.1038/s41391-023-00711-0, published online 07 September 2023

The following paragraph

Radiotracers that are approved by the Food and Drug Administration (FDA) for use in patients with PCa include carbon 11 (11C)-choline, fluorine 18 (18F)-sodium fluoride, 18F-fluciclovine (Axumin®, Blue Earth Diagnostics, Inc., Oxford, UK), gallium 68 (68Ga)-PSMA-11 (institutional use only in US), and 2-(3-{1-carboxy-5-[(6-18F-fluoropyridine-3-carbonyl)-amino]-pentyl}-ureido)-pentanedioic acid (18F-DCFPyL; PYLARIFY®, Progenics Pharmaceuticals, Inc. North Billerica, MA; US only).

should read:

Radiotracers that are approved by the Food and Drug Administration (FDA) for use in patients with PCa include carbon 11 (11C)-choline, fluorine 18 (18F)-sodium fluoride, 18F-fluciclovine (Axumin®, Blue Earth Diagnostics, Inc., Oxford, UK), gallium 68 (68Ga)-PSMA-11, and 2-(3-{1-carboxy-5-[(6-18F-fluoropyridine-3-carbonyl)-amino]-pentyl}-ureido)-pentanedioic acid (18F-DCFPyL; PYLARIFY®, Progenics Pharmaceuticals, Inc. North Billerica, MA). Access to radiotracers will continue to evolve, therefore, it is recommended to check regulatory authorities in specific countries for current approvals.

