# Supplementary material for: Application of next-generation imaging in biochemically recurrent prostate cancer
Source: Prostate Cancer Prostatic Dis. 2023 Sep 7;27(2):202–11. doi: 10.1038/s41391-023-00711-0 (PMC11096127; doi:10.1038/s41391-023-00711-0)
Supplement: Supplementary file 1 — Supplementary Appendix [file 41391_2023_711_MOESM1_ESM.docx]

**Table S1.** Levels of evidence for the included studies on the diagnostic and management changing utility of NGI in the BCR setting.

| ***Author (year)*** | ***Title*** | ***Evidence^*^*** |
| --- | --- | --- |
| Giovacchini et al. (2010) [1] | Predictive factors of [(11)C]choline PET/CT in patients with biochemical failure after radical prostatectomy | 2b |
| Jadvar et al. (2012) [2] | Prospective evaluation of ^18^F-NaF and ^18^F-FDG PET/CT in detection of occult metastatic disease in biochemical recurrence of prostate cancer | 1b |
| Roy et al. (2013) [3] | Comparative sensitivities of functional MRI sequences in detection of local recurrence of prostate carcinoma after radical prostatectomy or external-beam radiotherapy | 2b |
| Hillner et al. (2014) [4] | Impact of ^18^F-fluoride PET in patients with known prostate cancer: initial results from the National Oncologic PET Registry | 2b |
| Cha et al. (2015) [5] | Evaluation of suspected soft tissue lesion in the prostate bed after radical prostatectomy using 3T multiparametric magnetic resonance imaging | 1b |
| Hötker et al. (2015) [6] | Prostate Cancer: assessing the effects of androgen-deprivation therapy using quantitative diffusion-weighted and dynamic contrast-enhanced MRI | 2b |
| Chiaravalloti et al. (2016) [7] | PET/CT with ^18^F-choline after radical prostatectomy in patients with PSA≤ 2 ng/ml. Can PSA velocity and PSA doubling time help in patient selection? | 1b |
| Mapelli et al. (2016) [8] | 11C- or 18F-Choline PET/CT for Imaging Evaluation of Biochemical Recurrence of Prostate Cancer | 5 |
| Nanni et al. (2016) [9] | ^18^F-FACBC (anti1-amino-3-18F-fluorocyclobutane-1-carboxylic acid) versus ^11^C-choline PET/CT in prostate cancer relapse: results of a prospective trial | 1b |
| Dietlein et al. (2017) [10] | PSA-stratified performance of 18F-and 68Ga-PSMA PET in patients with biochemical recurrence of prostate cancer | 1b |
| Freitag et al. (2017) [11] | Local recurrence of prostate cancer after radical prostatectomy is at risk to be missed in (68)Ga-PSMA-11-PET of PET/CT and PET/MRI: comparison with mpMRI integrated in simultaneous PET/MRI | 2b |
| van Leeuwen et al. (2017) [12] | Prospective evaluation of 68Gallium-prostate-specific membrane antigen positron emission tomography/computed tomography for preoperative lymph node staging in prostate cancer | 1b |
| Yoon et al. (2017) [13] | Prostate-Specific Antigen and Prostate-Specific Antigen Kinetics in Predicting (18)F-Sodium Fluoride Positron Emission Tomography-Computed Tomography Positivity for First Bone Metastases in Patients with Biochemical Recurrence after Radical Prostatectomy | 2b |
| Gareen et al. (2018) [14] | Hospice admission and survival after ^18^F-fluoride PET performed for evaluation of osseous metastatic disease in the National Oncologic PET Registry | 2b |
| Hofman et al. (2018) [15] | Prostate-specific Membrane Antigen PET: Clinical Utility in Prostate Cancer, Normal Patterns, Pearls, and Pitfalls | 5 |
| Ost et al. (2018) [16] | Surveillance or Metastasis-Directed Therapy for Oligometastatic Prostate Cancer Recurrence: A Prospective, Randomized, Multicenter Phase II Trial | 1b |
| Siva et al. (2018) [17] | Stereotactic abative body radiotherapy (SABR) for oligometastatic prostate cancer: a prospective clinical trial | 1b |
| Alipour et al. (2019) [18] | Guiding management of therapy in prostate cancer: time to switch from conventional imaging to PSMA PET? | 5 |
| Andriole et al. (2019) [19] | The Impact of Positron Emission Tomography with ^18^F-Fluciclovine on the Treatment of Biochemical Recurrence of Prostate Cancer: Results from the LOCATE Trial | 1b |
| Calais et al. (2019) [20] | (18)F-fluciclovine PET-CT and (68)Ga-PSMA-11 PET-CT in patients with early biochemical recurrence after prostatectomy: a prospective, single-center, single-arm, comparative imaging trial | 1b |
| Fendler et al. (2019) [21] | Assessment of ^68^Ga-PSMA-11 PET Accuracy in Localizing Recurrent Prostate Cancer: A Prospective Single-Arm Clinical Trial | 1b |
| Ghafoor et al. (2019) [22] | Multimodality Imaging of Prostate Cancer | 5 |
| Giesel et al. (2019) [23] | Detection efficacy of ^18^F-PSMA-1007 PET/CT in 251 patients with biochemical recurrence of prostate cancer after radical prostatectomy | 2b |
| Gillessen et al. (2019) [24] | Management of Patients with Advanced Prostate Cancer: Report of the Advanced Prostate Cancer Consensus Conference | 5 |
| Mansbridge et al (2019) [25] | The use of MRI and PET imaging studies for prostate cancer management: brief update, clinical recommendations, and technological limitations | 5 |
| Pernthaler et al. (2019) [26] | A Prospective Head-to-Head Comparison of 18F-Fluciclovine With ^68^Ga-PSMA-11 in Biochemical Recurrence of Prostate Cancer in PET/CT | 2b |
| Raveenthiran et al. (2019) [27] | The use of (68)Ga-PET/CT PSMA to determine patterns of disease for biochemically recurrent prostate cancer following primary radiotherapy | 2b |
| Sandgren et al. (2019) [28] | Imaging for the detection of locoregional recurrences in biochemical progression after radical prostatectomy—a systematic review | 1a |
| Sheikhbahaei et al. (2019) [29] | (18)F-NaF-PET/CT for the detection of bone metastasis in prostate cancer: a meta-analysis of diagnostic accuracy studies | 1a |
| Barwick et al. (2020) [30] | Changing Landscape of Imaging in Recurrent Prostate Cancer | 5 |
| Eiber et al. (2020) [31] | (18)F-rhPSMA-7 PET for the Detection of Biochemical Recurrence of Prostate Cancer After Radical Prostatectomy | 2b |
| Ost et al. (2020) [32] | Surveillance or metastasis-directed therapy for oligometastatic prostate cancer recurrence (STOMP): Five-year results of a randomized phase II trial | 1b |
| Phillips et al. (2020) [33] | Outcomes of Observation vs Stereotactic Ablative Radiation for Oligometastatic Prostate Cancer: The ORIOLE Phase 2 Randomized Clinical Trial | 1b |
| Scarsbrook et al. (2020) [34] | Effect of ^18^F-Fluciclovine Positron Emission Tomography on the Management of Patients With Recurrence of Prostate Cancer: Results From the FALCON Trial | 1b |
| Sonni et al. (2020) [35] | Impact of ^68^Ga-PSMA-11 PET/CT on staging and management of prostate cancer patients in various clinical settings: a prospective single-center study | 2b |
| Tanaka et al. (2020) [36] | Current Imaging Techniques for and Imaging Spectrum of Prostate Cancer Recurrence and Metastasis: A Pictorial Review | 5 |
| Wurzer et al. (2020) [37] | Radiohybrid Ligands: A Novel Tracer Concept Exemplified by (18)F- or (68)Ga-Labeled rhPSMA Inhibitors | 5 |
| Farolfi et al. (2021) [38] | Positron Emission Tomography and Whole-body Magnetic Resonance Imaging for Metastasis-directed Therapy in Hormone-sensitive Oligometastatic Prostate Cancer After Primary Radical Treatment: A Systematic Review | 1a |
| Fendler et al. (2021) [39] | False positive PSMA PET for tumor remnants in the irradiated prostate and other interpretation pitfalls in a prospective multi-center trial | 1b |
| Glicksman et al. (2021) [40] | Curative-intent Metastasis-directed Therapies for Molecularly-defined Oligorecurrent Prostate Cancer: A Prospective Phase II Trial Testing the Oligometastasis Hypothesis | 2b |
| Kirste et al. (2021) [41] | Combining (68)Ga-PSMA-PET/CT-Directed and Elective Radiation Therapy Improves Outcome in Oligorecurrent Prostate Cancer: A Retrospective Multicenter Study | 2b |
| Kroeze et al. (2021) [42] | Prostate-specific Membrane Antigen Positron Emission Tomography-detected Oligorecurrent Prostate Cancer Treated with Metastases-directed Radiotherapy: Role of Addition and Duration of Androgen Deprivation | 2b |
| Jani et al. (2021) [43] | ^18^F-fluciclovine-PET/CT imaging versus conventional imaging alone to guide postprostatectomy salvage radiotherapy for prostate cancer (EMPIRE-1): a single center, open-label, phase 2/3 randomized controlled trial | 1b |
| Morris et al. (2021) [44] | Diagnostic Performance of ^18^F-DCFPyL-PET/CT in Men with Biochemically Recurrent Prostate Cancer: Results from the CONDOR Phase III, Multicenter Study | 1b |
| Pienta et al. (2021) [45] | A Phase 2/3 Prospective Multicenter Study of the Diagnostic Accuracy of Prostate Specific Membrane Antigen PET/CT with ^18^F-DCFPyL in Prostate Cancer Patients (OSPREY) | 1b |
| Abghari-Gerst et al. (2022) [46] | A Comprehensive Assessment of (68)Ga-PSMA-11 PET in Biochemically Recurrent Prostate Cancer: Results from a Prospective Multicenter Study on 2005 Patients | 1b |
| Checcucci et al. (2022) [47] | The real-time intraoperative guidance of the new HIFU Focal-One(®) platform allows to minimize the perioperative adverse events in salvage setting | 1b |
| De Man et al. (2022) [48] | (18)F-PSMA-11 Versus (68)Ga-PSMA-11 Positron Emission Tomography/Computed Tomography for Staging and Biochemical Recurrence of Prostate Cancer: A Prospective Double-blind Randomized Cross-over Trial | 1b |
| Dong et al. (2022) [49] | The European Association of Urology Biochemical Recurrence Risk Groups Predict Findings on PSMA PET in Patients with Biochemically Recurrent Prostate Cancer After Radical Prostatectomy | 2b |
| Ferdinandus et al. (2022) [50] | PSMA PET Validates Higher Rates of Metastatic Disease for European Association of Urology Biochemical Recurrence Risk Groups: An International Multicenter Study | 1b |
| Ferrari et al. (2022) [51] | [(18)F]fluciclovine vs. [(18)F]fluorocholine Positron Emission Tomography/Computed Tomography: A Head-to-Head Comparison for Early Detection of Biochemical Recurrence in Prostate Cancer Patients | 1b |
| Mena et al. (2022) [52] | Predictors of (18)F-DCFPyL PET/CT Positivity in Patients with Biochemical Recurrence of Prostate Cancer After Local Therapy | 1b |
| Olivier et al. (2022) [53] | Phase III study of (18)F-PSMA-1007 versus (18)F-fluorocholine PET/CT for localization of prostate cancer biochemical recurrence: a prospective, randomized, cross-over, multicenter study | 1b |
| Orevi et al. (2022) [54] | False Positive Findings of [(18)F]PSMA-1007 PET/CT in Patients After Radical Prostatectomy with Undetectable Serum PSA Levels | 2b |
| Schuster et al. (2022) [55] | Detection rate of ^18^F-rhPSMA-7.3 PET in patients with suspected prostate cancer recurrence: Results from a phase 3, prospective, multicenter study (SPOTLIGHT) | 1b |
| Ulaner et al. (2022) [56] | (18)F-DCFPyL PET/CT for Initially Diagnosed and Biochemically Recurrent Prostate Cancer: Prospective Trial with Pathologic Confirmation | 1b |

*Levels of evidence [57] pertinent to therapeutic, diagnostic, or prognostic studies: 1a = systematic review (with homogeneity) of RCTs, inception cohort studies, or level 1 diagnostic studies; clinical decision rule validated in different populations or with 1b studies from different clinical centers. 1b = individual RCT (with narrow confidence intervals), inception cohort study with > 80% follow‐up, or validating cohort study with good reference standards; clinical decision rule validated on a single population or tested within one clinical center. 1c = all or none study; absolute SpPins (Specific tests when Positive rules IN the disease) and SnNouts (Sensitive tests when Negative rules OUT the disease). 2a = systematic review (with homogeneity) of therapeutic cohort studies, retrospective cohort studies, untreated control groups in RCTs, or level >2 diagnostic studies. 2b = individual cohort studies (including low-quality RCTs), retrospective cohort study, follow‐up of untreated control patients RCTs, or exploratory cohort study with good reference standards; clinical decision rule validated on split‐sample only or databases. 2c = “outcomes” research. 3a = systematic review of case-control studies. 3b = individual case-control study, non‐consecutive study, or study without consistently applied reference standards. 4 = case series. 5 = expert opinion without explicit critical appraisal or based on physiology bench research or “first principles”.

# References

1. Giovacchini G, Picchio M, Coradeschi E, Bettinardi V, Gianolli L, Scattoni V, et al. Predictive factors of [(11)C]choline PET/CT in patients with biochemical failure after radical prostatectomy. Eur J Nucl Med Mol Imaging. 2010;37:301-309.

2. Jadvar H, Desai B, Ji L, Conti PS, Dorff TB, Groshen SG, et al. Prospective evaluation of 18F-NaF and 18F-FDG PET/CT in detection of occult metastatic disease in biochemical recurrence of prostate cancer. Clin Nucl Med. 2012;37:637.

3. Roy C, Foudi F, Charton J, Jung M, Lang H, Saussine C, et al. Comparative sensitivities of functional MRI sequences in detection of local recurrence of prostate carcinoma after radical prostatectomy or external-beam radiotherapy. AJR Am J Roentgenol. 2013;200:W361-368.

4. Hillner BE, Siegel BA, Hanna L, Duan F, Shields AF, Coleman RE. Impact of 18F-fluoride PET in patients with known prostate cancer: initial results from the National Oncologic PET Registry. J Nucl Med. 2014;55:574-581.

5. Cha D, Kim CK, Park SY, Park JJ, Park BK. Evaluation of suspected soft tissue lesion in the prostate bed after radical prostatectomy using 3T multiparametric magnetic resonance imaging. Magn Reson Imaging. 2015;33:407-412.

6. Hötker AM, Mazaheri Y, Zheng J, Moskowitz CS, Berkowitz J, Lantos JE, et al. Prostate Cancer: assessing the effects of androgen-deprivation therapy using quantitative diffusion-weighted and dynamic contrast-enhanced MRI. Eur Radiol. 2015;25:2665-2672.

7. Chiaravalloti A, Di Biagio D, Tavolozza M, Calabria F, Schillaci O. PET/CT with 18 F-choline after radical prostatectomy in patients with PSA≤ 2 ng/ml. Can PSA velocity and PSA doubling time help in patient selection? Eur J Nucl Med Mol Imaging. 2016;43:1418-1424.

8. Mapelli P, Incerti E, Ceci F, Castellucci P, Fanti S, Picchio M. 11C- or 18F-Choline PET/CT for Imaging Evaluation of Biochemical Recurrence of Prostate Cancer. J Nucl Med. 2016;57:43s-48s.

9. Nanni C, Zanoni L, Pultrone C, Schiavina R, Brunocilla E, Lodi F, et al. 18 F-FACBC (anti1-amino-3-18 F-fluorocyclobutane-1-carboxylic acid) versus 11 C-choline PET/CT in prostate cancer relapse: results of a prospective trial. Eur J Nucl Med Mol Imaging. 2016;43:1601-1610.

10. Dietlein F, Kobe C, Neubauer S, Schmidt M, Stockter S, Fischer T, et al. PSA-stratified performance of 18F-and 68Ga-PSMA PET in patients with biochemical recurrence of prostate cancer. J Nucl Med. 2017;58:947-952.

11. Freitag MT, Radtke JP, Afshar-Oromieh A, Roethke MC, Hadaschik BA, Gleave M, et al. Local recurrence of prostate cancer after radical prostatectomy is at risk to be missed in (68)Ga-PSMA-11-PET of PET/CT and PET/MRI: comparison with mpMRI integrated in simultaneous PET/MRI. Eur J Nucl Med Mol Imaging. 2017;44:776-787.

12. van Leeuwen PJ, Emmett L, Ho B, Delprado W, Ting F, Nguyen Q, et al. Prospective evaluation of 68Gallium-prostate-specific membrane antigen positron emission tomography/computed tomography for preoperative lymph node staging in prostate cancer. BJU Int. 2017;119:209-215.

13. Yoon J, Ballas L, Desai B, Jadvar H. Prostate-Specific Antigen and Prostate-Specific Antigen Kinetics in Predicting (18)F-Sodium Fluoride Positron Emission Tomography-Computed Tomography Positivity for First Bone Metastases in Patients with Biochemical Recurrence after Radical Prostatectomy. World J Nucl Med. 2017;16:229-236.

14. Gareen IF, Hillner BE, Hanna L, Makineni R, Duan F, Shields AF, et al. Hospice admission and survival after 18F-fluoride PET performed for evaluation of osseous metastatic disease in the National Oncologic PET Registry. J Nucl Med. 2018;59:427-433.

15. Hofman MS, Hicks RJ, Maurer T, Eiber M. Prostate-specific Membrane Antigen PET: Clinical Utility in Prostate Cancer, Normal Patterns, Pearls, and Pitfalls. Radiographics. 2018;38:200-217.

16. Ost P, Reynders D, Decaestecker K, Fonteyne V, Lumen N, De Bruycker A, et al. Surveillance or Metastasis-Directed Therapy for Oligometastatic Prostate Cancer Recurrence: A Prospective, Randomized, Multicenter Phase II Trial. J Clin Oncol. 2018;36:446-453.

17. Siva S, Bressel M, Murphy DG, Shaw M, Chander S, Violet J, et al. Stereotactic abative body radiotherapy (SABR) for oligometastatic prostate cancer: a prospective clinical trial. Eur Urol. 2018;74:455-462.

18. Alipour R, Azad A, Hofman MS. Guiding management of therapy in prostate cancer: time to switch from conventional imaging to PSMA PET? Ther Adv Med Oncol. 2019;11:1758835919876828.

19. Andriole GL, Kostakoglu L, Chau A, Duan F, Mahmood U, Mankoff DA, et al. The Impact of Positron Emission Tomography with 18F-Fluciclovine on the Treatment of Biochemical Recurrence of Prostate Cancer: Results from the LOCATE Trial. J Urol. 2019;201:322-331.

20. Calais J, Ceci F, Eiber M, Hope TA, Hofman MS, Rischpler C, et al. (18)F-fluciclovine PET-CT and (68)Ga-PSMA-11 PET-CT in patients with early biochemical recurrence after prostatectomy: a prospective, single-centre, single-arm, comparative imaging trial. Lancet Oncol. 2019;20:1286-1294.

21. Fendler WP, Calais J, Eiber M, Flavell RR, Mishoe A, Feng FY, et al. Assessment of 68Ga-PSMA-11 PET Accuracy in Localizing Recurrent Prostate Cancer: A Prospective Single-Arm Clinical Trial. JAMA Oncol. 2019;5:856-863.

22. Ghafoor S, Burger IA, Vargas AH. Multimodality Imaging of Prostate Cancer. J Nucl Med. 2019;60:1350-1358.

23. Giesel FL, Knorr K, Spohn F, Will L, Maurer T, Flechsig P, et al. Detection efficacy of 18F-PSMA-1007 PET/CT in 251 patients with biochemical recurrence of prostate cancer after radical prostatectomy. J Nucl Med. 2019;60:362-368.

24. Gillessen S, Attard G, Beer TM, Beltran H, Bjartell A, Bossi A, et al. Management of Patients with Advanced Prostate Cancer: Report of the Advanced Prostate Cancer Consensus Conference 2019. Eur Urol. 2020;77:508-547.

25. Mansbridge M, Chung E, Rhee H. The use of MRI and PET imaging studies for prostate cancer management: brief update, clinical recommendations, and technological limitations. Med Sci (Basel). 2019;7:85.

26. Pernthaler B, Kulnik R, Gstettner C, Salamon S, Aigner RM, Kvaternik H. A Prospective Head-to-Head Comparison of 18F-Fluciclovine With 68Ga-PSMA-11 in Biochemical Recurrence of Prostate Cancer in PET/CT. Clin Nucl Med. 2019;44:e566-e573.

27. Raveenthiran S, Yaxley J, Gianduzzo T, Kua B, McEwan L, Wong D, et al. The use of (68)Ga-PET/CT PSMA to determine patterns of disease for biochemically recurrent prostate cancer following primary radiotherapy. Prostate Cancer Prostatic Dis. 2019;22:385-390.

28. Sandgren K, Westerlinck P, Jonsson JH, Blomqvist L, Karlsson CT, Nyholm T, et al. Imaging for the detection of locoregional recurrences in biochemical progression after radical prostatectomy—a systematic review. Eur Urol Focus. 2019;5:550-560.

29. Sheikhbahaei S, Jones KM, Werner RA, Salas-Fragomeni RA, Marcus CV, Higuchi T, et al. (18)F-NaF-PET/CT for the detection of bone metastasis in prostate cancer: a meta-analysis of diagnostic accuracy studies. Ann Nucl Med. 2019;33:351-361.

30. Barwick TD, Castellucci P. Invited Commentary: Changing Landscape of Imaging in Recurrent Prostate Cancer. Radiographics. 2020;40:727-730.

31. Eiber M, Kroenke M, Wurzer A, Ulbrich L, Jooß L, Maurer T, et al. (18)F-rhPSMA-7 PET for the Detection of Biochemical Recurrence of Prostate Cancer After Radical Prostatectomy. J Nucl Med. 2020;61:696-701.

32. Ost P, Reynders D, Decaestecker K, Fonteyne V, Lumen N, Bruycker AD, et al. Surveillance or metastasis-directed therapy for oligometastatic prostate cancer recurrence (STOMP): Five-year results of a randomized phase II trial. J Clin Oncol. 2020;38:10.

33. Phillips R, Shi WY, Deek M, Radwan N, Lim SJ, Antonarakis ES, et al. Outcomes of Observation vs Stereotactic Ablative Radiation for Oligometastatic Prostate Cancer: The ORIOLE Phase 2 Randomized Clinical Trial. JAMA Oncol. 2020;6:650-659.

34. Scarsbrook AF, Bottomley D, Teoh EJ, Bradley KM, Payne H, Afaq A, et al. Effect of 18F-Fluciclovine Positron Emission Tomography on the Management of Patients With Recurrence of Prostate Cancer: Results From the FALCON Trial. Int J Radiat Oncol Biol Phys. 2020;107:316-324.

35. Sonni I, Eiber M, Fendler WP, Alano RM, Vangala SS, Kishan AU, et al. Impact of 68Ga-PSMA-11 PET/CT on staging and management of prostate cancer patients in various clinical settings: a prospective single-center study. J Nucl Med. 2020;61:1153-1160.

36. Tanaka T, Yang M, Froemming AT, Bryce AH, Inai R, Kanazawa S, et al. Current Imaging Techniques for and Imaging Spectrum of Prostate Cancer Recurrence and Metastasis: A Pictorial Review. Radiographics. 2020;40:709-726.

37. Wurzer A, Di Carlo D, Schmidt A, Beck R, Eiber M, Schwaiger M, et al. Radiohybrid Ligands: A Novel Tracer Concept Exemplified by (18)F- or (68)Ga-Labeled rhPSMA Inhibitors. J Nucl Med. 2020;61:735-742.

38. Farolfi A, Hadaschik B, Hamdy FC, Herrmann K, Hofman MS, Murphy DG, et al. Positron Emission Tomography and Whole-body Magnetic Resonance Imaging for Metastasis-directed Therapy in Hormone-sensitive Oligometastatic Prostate Cancer After Primary Radical Treatment: A Systematic Review. Eur Urol Oncol. 2021;4:714-730.

39. Fendler WP, Calais J, Eiber M, Simko JP, Kurhanewicz J, Santos RD, et al. False positive PSMA PET for tumor remnants in the irradiated prostate and other interpretation pitfalls in a prospective multi-center trial. Eur J Nucl Med Mol Imaging. 2021;48:501-508.

40. Glicksman RM, Metser U, Vines D, Valliant J, Liu Z, Chung PW, et al. Curative-intent Metastasis-directed Therapies for Molecularly-defined Oligorecurrent Prostate Cancer: A Prospective Phase II Trial Testing the Oligometastasis Hypothesis. Eur Urol. 2021;80:374-382.

41. Kirste S, Kroeze SGC, Henkenberens C, Schmidt-Hegemann NS, Vogel MME, Becker J, et al. Combining (68)Ga-PSMA-PET/CT-Directed and Elective Radiation Therapy Improves Outcome in Oligorecurrent Prostate Cancer: A Retrospective Multicenter Study. Front Oncol. 2021;11:640467.

42. Kroeze SGC, Henkenberens C, Schmidt-Hegemann NS, Vogel MME, Kirste S, Becker J, et al. Prostate-specific Membrane Antigen Positron Emission Tomography-detected Oligorecurrent Prostate Cancer Treated with Metastases-directed Radiotherapy: Role of Addition and Duration of Androgen Deprivation. Eur Urol Focus. 2021;7:309-316.

43. Jani AB, Schreibmann E, Goyal S, Halkar R, Hershatter B, Rossi PJ, et al. 18F-fluciclovine-PET/CT imaging versus conventional imaging alone to guide postprostatectomy salvage radiotherapy for prostate cancer (EMPIRE-1): a single centre, open-label, phase 2/3 randomised controlled trial. Lancet. 2021;397:1895-1904.

44. Morris MJ, Rowe SP, Gorin MA, Saperstein L, Pouliot F, Josephson D, et al. Diagnostic Performance of ^18^F-DCFPyL-PET/CT in Men with Biochemically Recurrent Prostate Cancer: Results from the CONDOR Phase III, Multicenter Study. Clin Cancer Res. 2021;27:3674-3682.

45. Pienta KJ, Gorin MA, Rowe SP, Carroll PR, Pouliot F, Probst S, et al. A Phase 2/3 Prospective Multicenter Study of the Diagnostic Accuracy of Prostate Specific Membrane Antigen PET/CT with ^18^F-DCFPyL in Prostate Cancer Patients (OSPREY). J Urol. 2021;206:52-61.

46. Abghari-Gerst M, Armstrong WR, Nguyen K, Calais J, Czernin J, Lin D, et al. A Comprehensive Assessment of (68)Ga-PSMA-11 PET in Biochemically Recurrent Prostate Cancer: Results from a Prospective Multicenter Study on 2,005 Patients. J Nucl Med. 2022;63:567-572.

47. Checcucci E, De Luca S, Piramide F, Garrou D, Mosca A, Galla A, et al. The real-time intraoperative guidance of the new HIFU Focal-One(®) platform allows to minimize the perioperative adverse events in salvage setting. J Ultrasound. 2022;25:225-232.

48. De Man K, Van Laeken N, Schelfhout V, Fendler WP, Lambert B, Kersemans K, et al. (18)F-PSMA-11 Versus (68)Ga-PSMA-11 Positron Emission Tomography/Computed Tomography for Staging and Biochemical Recurrence of Prostate Cancer: A Prospective Double-blind Randomised Cross-over Trial. Eur Urol. 2022;82:501-509.

49. Dong L, Su Y, Zhu Y, Markowski MC, Xin M, Gorin MA, et al. The European Association of Urology Biochemical Recurrence Risk Groups Predict Findings on PSMA PET in Patients with Biochemically Recurrent Prostate Cancer After Radical Prostatectomy. J Nucl Med. 2022;63:248-252.

50. Ferdinandus J, Fendler WP, Farolfi A, Washington S, Mohamad O, Pampaloni MH, et al. PSMA PET Validates Higher Rates of Metastatic Disease for European Association of Urology Biochemical Recurrence Risk Groups: An International Multicenter Study. J Nucl Med. 2022;63:76-80.

51. Ferrari C, Mammucci P, Lavelli V, Pisani AR, Nappi AG, Rubini D, et al. [(18)F]fluciclovine vs. [(18)F]fluorocholine Positron Emission Tomography/Computed Tomography: A Head-to-Head Comparison for Early Detection of Biochemical Recurrence in Prostate Cancer Patients. Tomography. 2022;8:2709-2722.

52. Mena E, Rowe SP, Shih JH, Lindenberg L, Turkbey B, Fourquet A, et al. Predictors of (18)F-DCFPyL PET/CT Positivity in Patients with Biochemical Recurrence of Prostate Cancer After Local Therapy. J Nucl Med. 2022;63:1184-1190.

53. Olivier P, Giraudet AL, Skanjeti A, Merlin C, Weinmann P, Rudolph I, et al. Phase III study of (18)F-PSMA-1007 versus (18)F-fluorocholine PET/CT for localization of prostate cancer biochemical recurrence: a prospective, randomized, cross-over, multicenter study. J Nucl Med. 2022:jnumed.122.264743.

54. Orevi M, Ben-Haim S, Abourbeh G, Chicheportiche A, Mishani E, Yutkin V, et al. False Positive Findings of [(18)F]PSMA-1007 PET/CT in Patients After Radical Prostatectomy with Undetectable Serum PSA Levels. Front Surg. 2022;9:943760.

55. Schuster D, SPOTLIGHT Study Group. Detection rate of 18F-rhPSMA-7.3 PET in patients with suspected prostate cancer recurrence: Results from a phase 3, prospective, multicenter study (SPOTLIGHT). J Clin Oncol. 2022;40:9-9.

56. Ulaner GA, Thomsen B, Bassett J, Torrey R, Cox C, Lin K, et al. (18)F-DCFPyL PET/CT for Initially Diagnosed and Biochemically Recurrent Prostate Cancer: Prospective Trial with Pathologic Confirmation. Radiology. 2022;305:419-428.

57. Howick J, Chalmers I, Glasziou P, Greenhalgh T, Heneghan C, Liberati A, et al. The Oxford Levels of Evidence 1. 2009. <http://www.cebm.net/oxford-centre-evidence-based-medicine-levels-evidence-march-2009>.
